# Let’s Play Games: A Comparison of Case-Based Learning Approach With Gamification Technique

**DOI:** 10.7759/cureus.27612

**Published:** 2022-08-02

**Authors:** Umema Zafar, Madiha Khattak, Hamna Zafar, Huma Rehman

**Affiliations:** 1 Physiology, Khyber Medical College, Peshawar, PAK; 2 Medicine, Khyber Medical College, Peshawar, PAK

**Keywords:** case-based learning, comparison, satisfaction, effectiveness, gamification technique

## Abstract

Case-based learning (CBL) has been in practice throughout the world for several decades now. Our institute adopted it some four years back when we shifted toward a modular system of teaching. It is the main technique being used for conducting small group discussions. We decided to introduce a new technique called the gamification technique for conducting small group discussions. There was a need to determine the effectiveness of this new method, as well as to assess factors for its preference so that it could be modified to increase its efficacy. The aim of this research was to quantitatively and qualitatively assess the effectiveness of said gamification teaching technique by comparison with the traditional CBL technique. This was a mixed-method, randomized controlled trial. It was conducted at Khyber Medical College on first-year medical students from June to October 2021. Group-based teaching involving both CBL and the gamification approach was used in this study in a crossover manner. Addressing the ethical concerns, and after informed consent pre-testing and post-testing were done to quantify the performance, an open-ended survey was disseminated after the sessions to check the perceptions of the students. The study recorded (quantitatively) that the post-testing mean score of the gamification teaching technique was 3.41 ± 0.982. For CBL, the mean was 3.55 ± 1.055. This showed a recording of an insignificant difference with a p-value of 0.608. In qualitative analysis, about 12 (80%) students preferred the gamification technique. Their perception was that it instilled competitiveness and increased the involvement of students in class. Gamification also raised their motivation level. This research further revealed that the CBL approach had the advantage of quick learning via the facilitator presentation. Due to the handouts, it was easy to follow. One of the negative points of CBL was that the participants found it a boring and monotonous way of learning. The chief drawback of gamification was that the students were unsure about the accuracy of the information they initially prepared, as it was not being directed by the facilitator. The study concluded an insignificant quantitative difference between the two techniques. On the qualitative end, however, the students preferred gamification.

## Introduction

A different and relatively new technique recently introduced in medical education is gamification. Muntasir et al. define gamification as “a process of enhancing services with motivational affordances borrowed from games to invoke behavioral outcomes.” The aim of modifying traditional teaching techniques to unconventional new ones should be to modify behavior to promote better and more active learning [1]. One way of behavior modification is the reward system, either in the form of material rewards or personal satisfaction. Another important component of behavior modification and the learning process is timely feedback [2]. The gamification technique is a possible means of enhancing active learning and affecting behavior positively. Two educators in Australia, Gemeah Howarth-Hockey and Peter Stride, have been following various games in medical education, such as “you too can become a physician” and “Medi-team challenge.” The responses to these two games have been quite encouraging [3]. According to Ahmadi et al., the benefits of gamification include increased participation and enhanced productivity, efficiency, and creativity [4].

The gamification trend dates back to the 1980s, but its application in education has accelerated from 2010 onwards, and this technique for learning and teaching has only recently been adopted in medical education [5,6]. Gamification has been applied to the study of clinical cases by creating online simulations, tests, and reward systems. One example is Synaptix, a web-based platform based on gamification techniques [7]. MedSense is another such platform, where students can see and learn from case-based simulations and share interesting cases with fellow students as well as teachers [8]. Huang et al. found in a quasi-experimental study that a gamified group had more out-of-class discussion activities than the control group. Moreover, the students expressed that the gamification method was fun and engaging for the learners and it enhanced motivation [9].

Two types of gamification techniques described in the literature are challenge-based and immersion-based techniques [10]. We chose the challenge-based gamification technique, as it incorporates the feature of competitive learning; the participants learn by being challenged [11]. In contrast, the immersion-based gamification technique involves creating a learning environment that immerses the student in the learning process [12].

The case-based learning (CBL) method utilizes the problem-based learning (PBL) principles. In CBL, the students are given a reading task, then a problem is presented, additional reading is done, and then there is a discussion in the presence of a facilitator. In PBL, a problem is presented, which is then explored, additional reading is done, and then there is a discussion [13]. In our institute, this is then followed by an additional last step, namely, a question and answer session conducted by the facilitator. This type of CBL technique has been adopted in our institute, as it was advocated for by the Department of Medical Education, due to the feasibility of its application in our setup as well as the learning benefits.

In his 2016 paper, McLean defines CBL as a teaching technique that utilizes clinical vignettes to link theory to clinical practice and stimulates curiosity in students [14].

Although the gamification method seems to be more interesting and successful in terms of assessment scores, there is a need to assess the effectiveness and the satisfaction status of the learners related to these two methodologies. What is unique about this research is that the effectiveness of both techniques was gauged with a larger group. Usually, small group discussion (SGD) is undertaken in small groups of eight to 15 participants. We dealt with groups consisting of 60-65 students. However, the technique was applied with some modifications of subgrouping and task division.

This research aims to compare the effectiveness of the gamification technique in conducting small group teaching with the CBL format currently being used in our institute. We also aimed to gauge the perception and preference of the students regarding the two techniques.

The manuscript has been presented at the Experimental Biology 2022, Philadelphia, United States, as a paper presentation as well as a poster. Its abstract has been published in the FASEB journal as a conference proceeding.

## Materials and methods

This is a mixed-method educational trial, conducted at Khyber Medical College (KMC) in Peshawar. It was further augmented by a survey of the perceptions of the participants.

The total sample in each group depended on the attendance of the students, and there were four groups. Undergraduate students in their first year of Bachelor of Medicine, Bachelor of Surgery (MBBS) at KMC who were present in class during the sessions were included.

Pre-tests were done in a large-group-format (LGF) lecture a week before either of the techniques was implemented in each session. The pre-test consisted of three multiple-choice questions (MCQs) and two true/false questions (a total of five questions per test per session). A similar method/format was adopted for the post-tests, which were done in an LGF within one to two days after each session.

**Figure 1:**
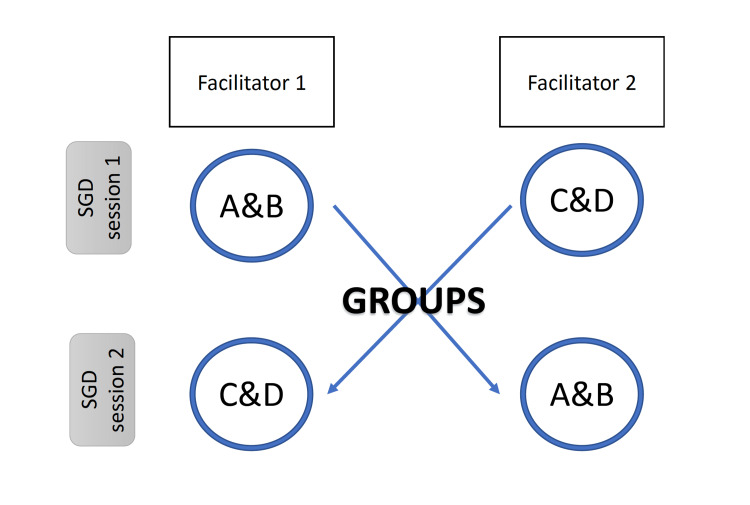
Crossover design of groups taken by the facilitators in the two sessions SGD: small group discussion.

**Figure 2:**
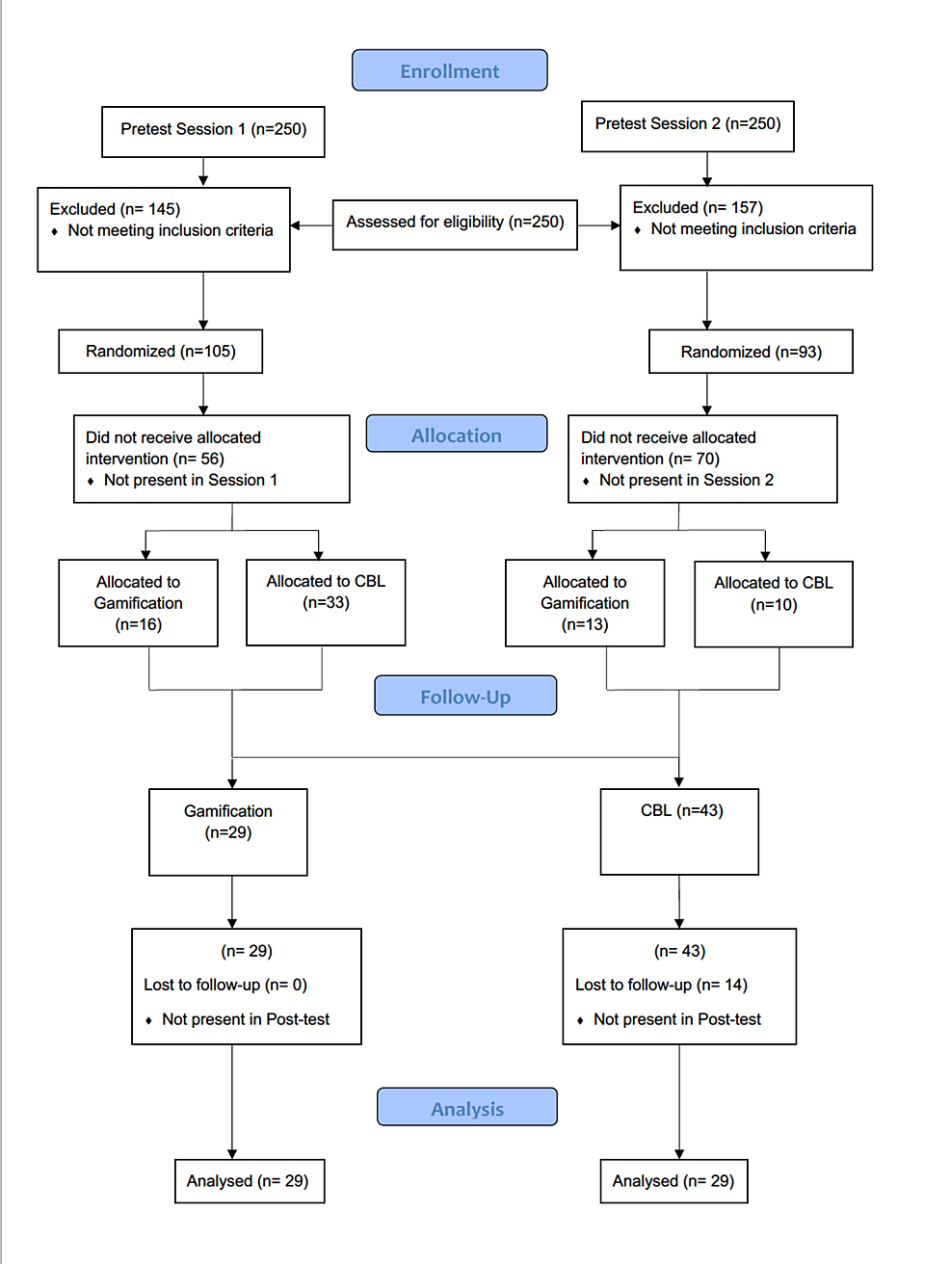
CONSORT flow diagram CONSORT: Consolidated Standards of Reporting Trials; CBL: case-based learning.

The technique applied to each group was randomly chosen for the first session. Due to the crossover design, for the second session, the technique was reversed for each group (Figure 1). The sampling technique adopted was simple random sampling within each group. The students in the groups were divided into subgroups via the lottery method too. Students who were absent on the day of the sessions were excluded. Allocations were concealed; neither the researcher nor the participants knew at the time of pre-testing which group of students would be randomized for which interventions (Figure 2).

The MBBS class of 250 students was divided into four groups, and two sessions were conducted separately for each group. Informed consent was obtained from all participants. Although they were not informed about the type of intervention (CBL or gamification) they would have during the SGD session beforehand, the blinding was irrelevant, as it was easy to guess which technique it was. The topic for the first session was “disorders of the neuromuscular junction” and for the second session, it was “comparison of smooth muscle contraction and skeletal muscle contraction.” These topics were considered because they were included in the curriculum at the time of the data collection/study period. In both sessions, two facilitators each took two groups. Facilitator 1 used the traditional CBL technique and facilitator 2 used the gamification technique. The traditional approach is the one normally followed at KMC. The two groups taken by facilitator 1 in the first session were then taken by facilitator 2 in the second session (Figure 1). The experience and qualifications of both facilitators are similar. Ultimately, a survey questionnaire constituting 10 open-ended questions was emailed to the students who attended the sessions (Appendix).

Case-based learning technique

**Figure 3:**
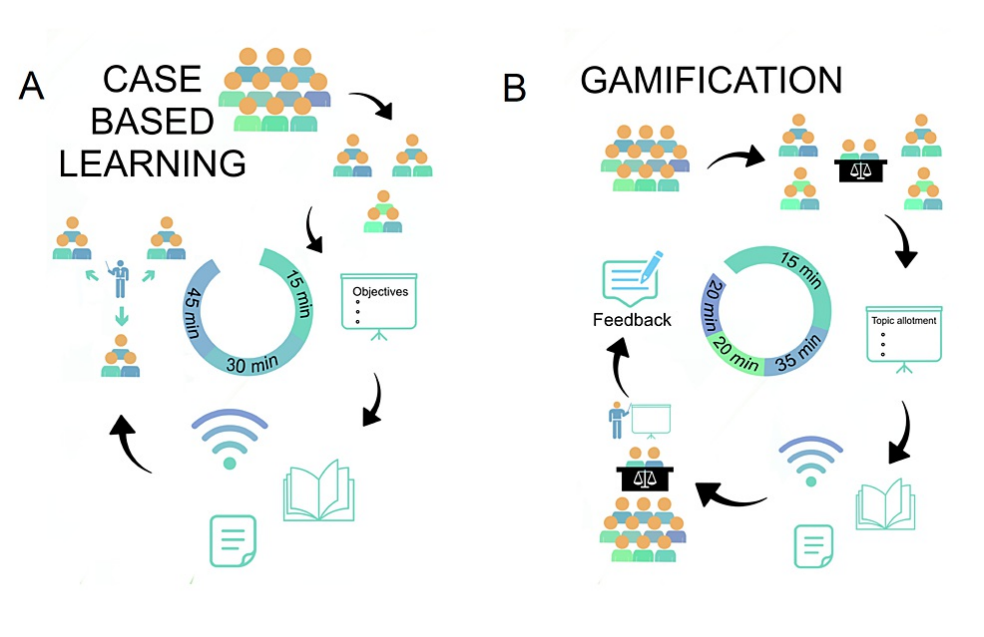
(A) Method for the case-based learning technique. (B) Method for the gamification technique

The students in each group were divided into subgroups of five to six each. The clinical scenario was portrayed on multimedia. The objectives to be met through discussions in the small groups were written on the whiteboard. The subgroups were briefed on the clinical scenario and instructed to discuss the objectives. The students were informed that they would all be asked questions by the facilitator, hence, individual participation was compulsory. This entire process took 15 minutes. Thereafter, they were given 30 minutes to discuss the topic, read textbooks, or search the internet. Handouts were also distributed. During this half hour, the students had a discussion under the watchful eyes of the facilitator. Thereafter, the facilitator went around and asked each group questions. CBL is not competitive because it is on a one-to-one basis and each student has to draw on their own prior knowledge. In contrast, with gamification, teams are formed and compete against each other based on their theoretical knowledge and understanding of the concepts (Figure 3A).

Gamification technique

This technique involved dividing the group into two subgroups and modifying teaching such that it became a competitive game. The main group was divided into two subgroups (group 1 and group 2), with an almost equal number of males and females seated in a circle. One judge was selected from each group. Each subgroup was then again subdivided, depending on the total strength of the class. A portion of the topic was allotted to each smaller subgroup for presentation. Once the topic had been allotted, the students had 35 minutes to study the topic and prepare presentations. During this time, the facilitator was there to guide the students, who studied from either textbooks or the internet. After this, there were a total of four rounds with two oral presentations per round. The duration of the presentations was two minutes. The judges then had a minute to write their decisions and comments on paper slips, which were collected. Hence, 20 minutes were required for the presentations and judging. The role of the judges was to study all the allotted topics and then judge the presenters from each group. The judgment was based upon presentation skills, observation of the time limit, relevancy, fluency, and clarity. The smaller subgroups prepared and presented their topics, and these presentations helped everyone to learn about all the topics. Everyone listened critically as the judgment was involved. At the end of the presentations, the judging slips were collected from the judges, and the results were written on the whiteboard. The comments of the judges were also read out, without revealing the judges’ identities, to provide feedback (Figure 3B).

Qualitative survey

A qualitative survey was conducted among all the students who attended all the sessions and were present at the time of the tests (a total of 29). They were asked 10 questions related to the two teaching techniques. They submitted answers either via email or in person on paper.

Analysis was performed using SPSS version 26.0 (IBM Corp., Armonk, NY). Mean ± standard deviation was calculated for numerical variables (pre- and post-test marks) and an unpaired t-test was applied. For the survey, thematic analysis of the data was done manually to identify codes, categories, and themes. The themes were analyzed after coding and a theory was derived.

## Results

A total of 29 students (16 in session 1 and 13 in session 2) attended the gamification-based SGDs and 43 (33 in session 1 and 10 in session 2) attended the CBL SGDs. The post-testing was done in a lecture-based class after the SGDs, and only the 58 students who had attended the actual SGD intervention were selected for post-testing analysis. Similarly, pre-testing was done in two lecture-based classes before the start of SGD interventions. A total of 46 students who attended the SGD intervention were analyzed for pre-testing.

There was no significant difference between the two techniques on the basis of the post-testing (p > 0.05). Similarly, there was also an insignificant difference between the two techniques in the pre-testing (p > 0.05).

**Figure 4:**
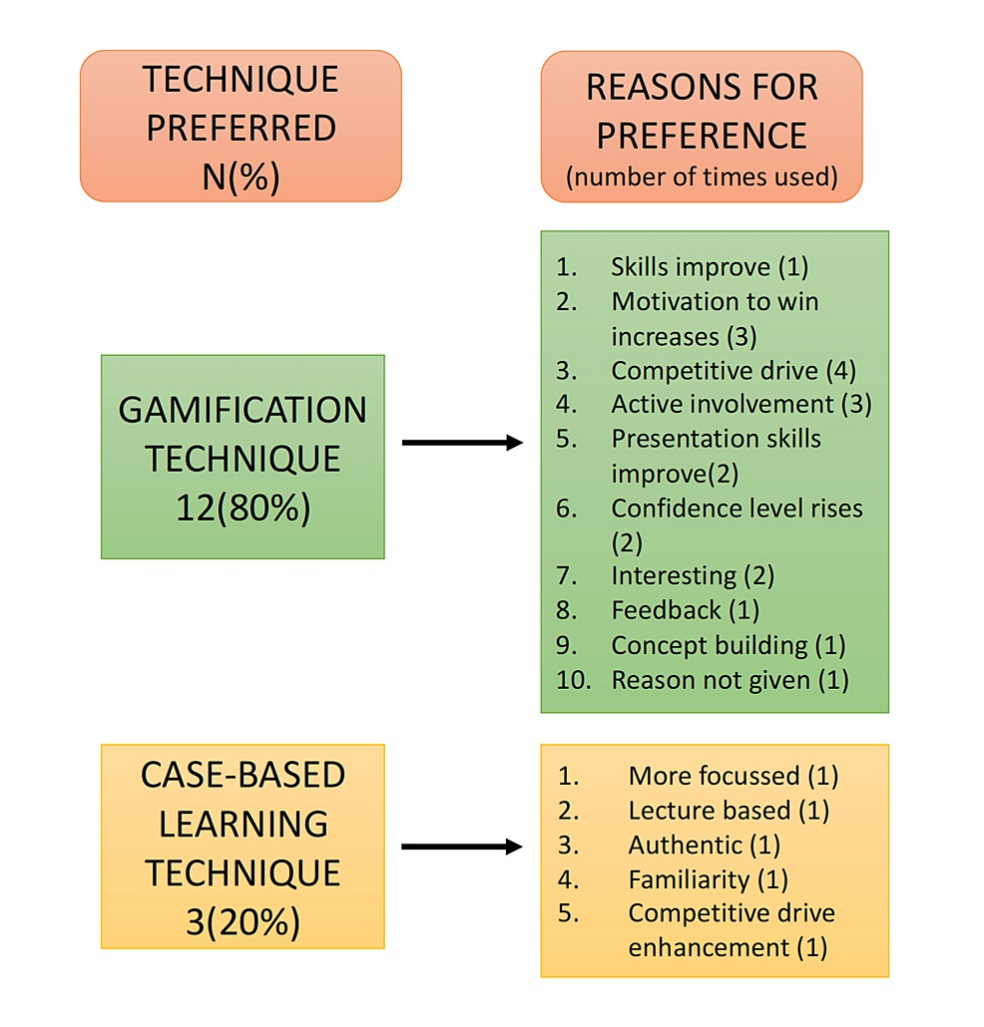
The technique preferred by the students and the reasons

**Figure 5:**
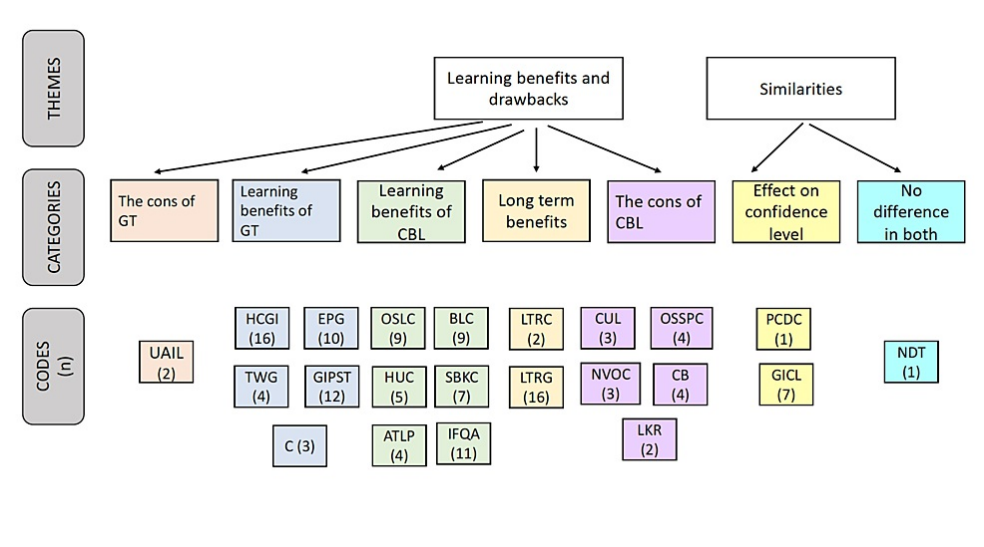
Thematic analysis of survey questionnaire GT: gamification technique; CBL: case-based learning; HCGI: healthy competition in gamification encouraged involvement; TWG: teamwork in gamification; EPG: encouraged participation in gamification; GIPST: gamification improved presentation in a short time; C: concept building; OSLT: on the spot learning with traditional; HUC: handouts useful in CBL; BLC: brief lecture in CBL; SBKC: sufficient basic knowledge imparted in CBL; ATLP: ample time to learn and present; IFQA: improved focus due to Q&A session; GD: group discussion; LTRG: long-term retainment in gamification more; LTRC: long-term retainment in CBL more; UAIL: uncertainty about the accuracy of information learned; CUL: CBL was the usual way of learning; NVOC: not very organized in CBL; OSSPC: only some students presented in CBL; CB: CBL was boring; LKR: low knowledge retainment; PCDC: presenters of CBL developed confidence; GICL: gamification method improved confidence level; NDT: no difference in the two techniques.

Out of the 29 students to whom the questionnaires were distributed, 15 responded. The response rate was calculated to be 51.72%. The thematic analysis of the open-ended survey resulted in the deduction of three main themes related to the two techniques, namely, preference, similarities, and learning benefits and drawbacks. The theory derived from this present research was that, although the gamification technique is preferred by the students, the techniques are equally beneficial in terms of academic performance (Figures 4, 5 and Table 1).

**Table 1:** Codes and abbreviations used for thematic analysis CBL: case-based learning; Q&A: question and answer.

S. No.	Codes	Abbreviations	Number of times used
1	Healthy competition in gamification encouraged involvement	HCGI	16
2	Teamwork in gamification	TWG	4
3	Encouraged participation in gamification	EPG	10
4	Gamification improved presentation in a short time	GIPST	12
5	Concept building	C	3
6	On-spot learning with traditional	OSLT	9
7	Handouts useful in CBL	HUC	5
8	Brief lecture in CBL	BLC	9
9	Sufficient basic knowledge imparted in CBL	SBKC	7
10	Ample time to learn and present	ATLP	4
11	Improved focus due to Q&A session	IFQA	11
12	Group discussion	GD	2
13	Long-term retainment in gamification more	LTRG	16
14	Long-term retainment in CBL more	LTRC	2
15	Uncertainty about the accuracy of information learned	UAIL	2
16	CBL was the usual way of learning	CUL	3
17	Not very organized in CBL	NVOC	3
18	Only some students presented in CBL	OSSPC	4
19	CBL was boring	CB	4
20	Low knowledge retainment	LKR	2
21	Presenters of CBL developed confidence	PCDC	1
22	Gamification method improved confidence level	GICL	7
23	No difference between the two techniques	NDT	1

## Discussion

The pre-testing was done to assess and compare the baseline knowledge of the students, and it showed no significant difference between the two methods of teaching. An insignificant difference in the scores removed the bias of prior knowledge of the topic. The post-test showed that the two techniques had similar results, based on the performance of the participants. Likewise, on one hand, a study conducted in 2010 compared the game-based learning technique with traditional CBL and showed that there was no difference (p > 0.05) between the two techniques, based on the post-intervention assessment questionnaire [15]. On the other hand, in 2019, Mackavey introduced an intervention that incorporated several game elements in a family nurse practitioner program in Texas. He introduced a gamified case-based technique in one semester and then compared the assessments of that semester with those of the previous semester. He found a significant improvement in the performance of the students in the gamified semester (p < 0.001) [16].

A majority (80%) of the participants preferred the gamification technique to the CBL in the present research. The students perceived that the two methods similarly focused on confidence-building among the participants. Improvements in confidence levels and the engagement of students in the teaching session were gauged by Raju et al., who used online platforms to transform learning and assessment into a gamified format. The study showed that over a full semester, student attendance and the degree of participation slowly improved [17]. According to the students, gamification was perceived to be more beneficial, as it promotes teamwork, improves timed presentation skills, and creates an environment of healthy competition, motivating them to give their best. In their 2019 study, Singhal et al. wrote that mild to moderate levels of stress induced by a healthy competitive pedagogical environment can promote learning and retention of information. However, the greatest challenge of the application of this technique is managing the stress levels induced by the competition [18]. Teamwork and a competitive environment must go hand in hand to prevent the stress from becoming overwhelming, and the role of the facilitator is important in this context. They must manage the session so that the flow is maintained and ambiguities are minimized [19]. Another benefit of gamification in the present study was the perceived long-term knowledge retention by the participants. According to constructivist learning theory, supporting learning through prompts, frequent checks, rewards or winning, and fear of losing can all enhance concentration levels and, hence, promote the learning process and knowledge retention [20]. The present study further revealed that the CBL approach has the advantage of rapid learning through presentations by the facilitator, which were easy to follow due to the handouts. The participants reported that they found CBL to be boring and monotonous. It lacked the excitement of intergroup competition, and the participants perceived it as disorganized.

The reported drawback of gamification was that the students were unsure of the accuracy of the information they prepared initially, as it was not directed by the facilitator. If the topic is boring but has many aspects, gamification should be applied to maintain the students’ interest and enhance long-term retention. If the topic is more clinically oriented and requires a holistic approach of combining basic sciences with clinical sciences, CBL should be used.

This study compared the novel gamified teaching technique with the traditional case-based approach. Some of the limitations we encountered were the poor attendance of students due to the current coronavirus disease 2019 (COVID-19) crisis, and the on-off shifting between on-campus and online teaching formats. The low student attendance led us to modify the interventions according to a lower strength batch of the students. Therefore, the statistical analysis and data processing had to be modified accordingly. However, this is a robust intervention, and it is recommended that it be reproduced in a different setup to gauge the veracity.

## Conclusions

Although many new and improved techniques are being developed and incorporated into undergraduate teaching systems, the case for the CBL technique remains strong, especially for medical students. Hence, both methods should be applied in undergraduate teaching, depending on the topic to be studied. Despite insignificant differences in the scores of the participants, the gamification technique was not only appreciated but also preferred by the students. Some of the plus points of the gamification technique were individual involvement, healthy competition, confidence boosting, and improvement of presentation skills.

## Appendices

**Table 2:** Questions included in the survey questionnaire CBL: case-based learning.

S. No.	Questions
1	How would you describe your experience with the gamification form of learning with facilitator 2?
2	How would you describe your experience with the CBL form of learning with facilitator 1?
3	What was the difference between the two methodologies?
4	Which method would you prefer and why?
5	How has gamification with facilitator 2 impacted your learning?
6	How has CBL with facilitator 1 impacted your learning?
7	Which aspect of the traditional learning method with facilitator 1 do you think had the most impact on your learning?
8	Which aspect of the gamification method with facilitator 2 do you think had the most impact on your learning?
9	Which type of method will help you remember the topic 10 years from now?
10	Would you like to add anything else before I conclude?
